# Application effect of initiation of enteral nutrition at different time periods after surgery in neonates with complex congenital heart disease: A retrospective analysis

**DOI:** 10.1097/MD.0000000000024149

**Published:** 2021-01-08

**Authors:** Na Du, Yanqin Cui, Wanhua Xie, Caixin Yin, Chen Gong, Xiuchun Chen

**Affiliations:** a CICU; b Outpatient Department; c Nursing Department, Guangzhou Women and Children's Medical Center, Guangzhou Medical University, Guangzhou, China.

**Keywords:** complex congenital heart disease, early enteral nutrition, feeding intolerance, nutritional status, neonates

## Abstract

Early enteral nutrition (EN) promotes the recovery of critically ill patients, but the initiation time for EN in neonates after cardiac surgery remains unclear.

This study aimed to investigate the effect of initiation time of EN after cardiac surgery in neonates with complex congenital heart disease (CHD).

Neonates with complex CHD admitted to the CICU from January 2015 to December 2017 were retrospectively analyzed. Patients were divided into the 24-hour Group (initiated at 24 hours after surgery in 2015) (n = 32) and 6-hour Group (initiated at 6 hours after surgery in 2016 and 2017) (n = 66). Data on the postoperative feeding intolerance, nutrition-related laboratory tests (albumin, prealbumin, retinol binding protein), and clinical outcomes (including duration of mechanical ventilation, CICU stay, and postoperative hospital stay) were collected.

The incidence of feeding intolerance was 56.3% in 24-hour Group and 39.4%, respectively (*P* = .116). As compared to 24-hour Group, prealbumin and retinol binding protein levels were higher (160.7 ± 64.3 vs 135.2 ± 28.9 mg/L, *P* = .043 for prealbumin; 30.7 ± 17.7 vs 23.0 ± 14.1 g/L *P* = .054 for retinol-binding protein). The duration of CICU stay (9.4 ± 4.5 vs 13.3 ± 10.4 day, *P* = .049) and hospital stay (11.6 ± 3.0 vs 15.8 ± 10.3 day, *P* = .028) were shorter in 6-hour Group.

Early EN improves nutritional status and clinical outcomes in neonates with complex CHD undergoing cardiac surgery, without significant feeding intolerance.

## 1 Introduction

Congenital heart disease (CHD) is the most common type of congenital defect.^[1]^ The incidence is approximately 4‰ to 10‰.^[2]^ Children with CHD are at risk of malnutrition. Their birth weight may be normal, but malnutrition and growth difficulties may occur after birth.^[3]^ In 2014, a study showed that CHD children under 2 years of age had a higher incidence of acute and chronic malnutrition, which was 51.2% and 40.5%, respectively.^[4]^ Increasing evidence suggests that malnutrition can prolong the time of mechanical ventilation and intensive care and increase the incidence of infection, complications, and death.^[5,6]^ As the nutritional status during the perioperative period directly affects the prognosis of children,^[7,8]^ perioperative nutritional support is particularly important. Although parenteral nutrition is an effective nutritional support method, its long-term use results in different degrees of complications.^[9]^ The guidelines recommend that enteral nutrition (EN) should be p if the child is tolerant.^[10]^ Studies have shown that early EN contributes to the development and maturation of gastrointestinal mucosa, which can promote the recovery of patients and reduce mortality.^[11,12]^ Nutrient intake and feeding tolerance are critical and affect the nutritional status of children.^[13]^ The symptoms of complex CHD, if not treated in time, quickly worsen, leading to an early death. To save lives, surgical intervention in the neonatal period is needed.^[14]^ Due to hemodynamic changes in children with CHD, mesenteric hypoperfusion may occur, resulting in feeding intolerance,^[7]^ which is particularly common among neonates. Moreover, the neonatal period is an important stage of development, and its metabolism has its own characteristics, needing to meet not only the postoperative energy expenditure but also growth and developmental needs. Therefore, attention to the energy supply of children undergoing surgery during the neonatal period should be noted. Moreover, it also puts higher requirements on perioperative monitoring and nutritional support.

At present, no consensus exists for the optimum time to initiate early EN support, which is generally considered as within 48 hours.^[15]^ Enhanced recovery after surgery (ERAS) recommends that EN should be initiated 6 hours after surgery based on the effective prevention of postoperative intestinal paralysis, nausea, and vomiting, which has been proved to have certain benefits for pediatric patients undergoing surgery.^[16,17]^ The objective of this study was to investigate the application effect of initiation of EN at different times after surgery in neonates with complex CHD to provide basis for the initiation time of EN.

## 2 Methods

### 2.1 Participants

The case data of neonates with complex CHD admitted to the cardiac intensive care unit (CICU) of Guangzhou Women and Children's Medical Center from January 2015 to December 2017 were retrospectively collected. The patients were initiated EN at 24 hours after surgery in the period between January and December 2015 (24-hour Group) and at 6 hours after surgery in the period between January 2016 to December 2017 (6-hour Group). The inclusion criteria were neonates; and complex CHD children with Risk Adjustment for Congenital Heart Surgery (RACHS) category 3-4 (Table 1).^[18]^ The exclusion criteria were as follows: premature infants; children with coarctation of the aortic or left ventricular outflow tract obstruction who had gastrointestinal ischemia before surgery^[19]^ and the initiation of early EN may result in ischemia and necrosis of intestinal mucosa; children with severe metabolic diseases; children with severe gastrointestinal diseases; children undergoing hemodialysis; children receiving extracorporeal membrane oxygenation; and children with incomplete data. This was a single-centered study approved by the ethics committee of the Guangzhou Women and Children's Medical Center.

**Table 1:** The RACHS categories, surgical procedures, and diagnosis of the 24-h and 6-h feeding groups.

Group	RACHS	Procedure name	Diagnoses
24-h group (n = 32)	3 (n = 23)	ASO (n = 19);	TGA + PDA (n = 19
		Systemic to pulmonary artery shunt (n = 1);	ccTGA (n = 1)
		RV to pulmonary artery conduit (n = 3);	PA + VSD (n = 1); PA + ASD + PDA (n = 2)
	4 (n = 9)	ASO with VSD closure (n = 3);	TGA + VSD (n = 3)
		Repair of TAPVC at age ≤30 d (n = 5);	TAPVC (n = 5)
		Repair of IAA with VSD closure (n = 1)	DORV + IAA + VSD (n = 1)
6-h group (n = 66)	3 (n = 38)	ASO (n = 27)	TGA + PDA (n = 26); TGA + ASD (n = 1)
		Systemic to pulmonary artery shunt (n = 6)	TGA + VSD + PS (n = 1); PA + PDA (n = 1); PA + RV dysplasia (n = 1); PA + VSD (n = 2); Ebstein anomaly + PDA (n = 1)
		ASO, pulmonary outflow tract augmentation (n = 1)	TGA + RVOT stenosis (n = 1)
		RV to pulmonary artery conduit (n = 2):	PA + ASD + PDA (n = 2)
		Reimplantation of anomalous right pulmonary artery (n = 2)	Anomalous right pulmonary artery arising from the ascending aorta (n = 2)
	4 (n = 28)	Repair of TAPVC at age ≤30 d (n = 25)	TAPVC (n = 25)
		ASO with VSD closure (n = 3)	TGA + VSD (n = 3)

### 2.2 Study design

EN was initiated at 24 and 6 hours after surgery, respectively, in the 24-hour Group and 6-hour Group during the different time periods due to the practice change in our center. The initial dose for both groups was 1 mL/(kg · h), and milk was added once every 6 h at the amount of 1 mL/kg until it reached the target calorie of 100 kcal/(kg · d).^[20,21]^ The initiation procedure was as follows: Based on the target calorie, children's weight, and energy density of the milk powder, the doctors calculated the target milk amount, and the nurses performed it according to the doctor's advice. After the initiation of EN, the feeding intolerance of the children was observed; related studies revealed no consensus for the definition of feeding intolerance, which could generally be assessed by gastric retention, abdominal distension, and vomiting.^[22,23]^ Gastric residual volume (GRV) exceeding 50% of the previous feeding volume for 2 consecutive times^[23]^ was defined as gastric retention. Residual volume referred to the volume of undigested contents obtained through the nasogastric feeding tube before the next feeding.^[22]^ The nurses conducted regular monitoring of the GRV. The patients underwent mechanical ventilation and nasal feeding, which continued for 3 hours each time, and the GRV was assessed by the injection pump-back of the gastric residues using a 20-mL syringe. If the children showed feeding tolerance, milk was added every 6 hours at an amount of 1 mL/kg until it reached the target milk amount. If the children showed feeding intolerance, milk was not temporarily added until the symptoms improved. If the children still showed feeding intolerance, the assessment continued after discontinuing feeding for 1 hour. If the children still showed feeding intolerance after discontinuing feeding for 1 hour, the nutritional department was consulted, and temporary fasting or symptomatic treatment was recommended. In the entire procedure, medical orders were made by the doctors and strictly performed by the nurses, and close observation was conducted. Once symptoms of severe feeding intolerance, such as gastrointestinal bleeding and intestinal obstruction, were observed, EN was immediately discontinued. The consultation with gastrointestinal physicians was performed for symptomatic treatments to ensure the safety of children.

The assessment indicators of feeding intolerance included the following: gastric retention: GRV exceeding 50% of the previous feeding volume for 2 consecutive times; repeated vomiting: vomiting for more than three times within 24 hours; and abdominal distension persisting for 24 hours or more.

The laboratory indicators included the following: albumin, prealbumin, and retinol-binding protein (RBP) and measured before operation and when discharged from the CICU.

Other nutritional and clinical outcome indicators included the following: weight; postoperative use of prokinetic drug (domperidone; postoperative ventilator-assisted ventilation time; observation window time from the initiation of EN to evacuation of the ventilator; postoperative staying time at CICU; length of hospital stay (pre- and postoperative stay in CICU, and hospital stay); and the occurrence of ventilator-associated pneumonia; hospitalization expenses.

### 2.3 Data collection

The medical records were reviewed, and demographic and clinical data were retrospectively collected. According to the objective description of children's conditions in the nursing records, their feeding intolerance during CICU monitoring was determined.

### 2.4 Statistical analysis

Statistical analyses were performed using the SPSS 22.0 software. The categorial data were expressed as n (%); the Chi-square test was used. The continuous data were expressed as mean ± SD, and the *t* test was used. A *P* value < .05 indicated statistical significance.

## 3 Results

### 3.1 Comparison of general conditions between the 2 groups

In the 2 groups, 97 patients were admitted to the CICU before surgery except for 1 who was transferred from the ward to the operating room directly. There was 1 patient with preoperative feeding intolerance in each group, but no prokinetic drugs were administered. No difference was found in the aforementioned indicators between the 2 groups (Table 2).

**Table 2:** Comparison of general conditions between the 2 groups.

Item		24-h group	6-h group	*P*
Sex (n)	Male	21 (65.6%)	52 (78.8%)	.161
	Female	11 (34.4%)	14 (21.2%)	
Age at surgery, d		11.3 ± 9.0	14.0 ± 8.0	.149
Grading of surgical risk (n)	Grade 3	23 (71.9%)	38 (57.6%)	.171
	Grade 4	9 (28.1%)	28 (42.4%)	
Cardiopulmonary bypass (n)		31 (96.9%)	62 (93.9%)	.897
Weight in CICU, kg		3.3 ± 0.6	3.2 ± 0.5	.568
Preoperative length of stay, d		3.9 ± 2.9	4.2 ± 4.4	.746
Preoperative feeding intolerance (n)		1 (3.1%)	1 (1.5%)	.549
Postoperative Vasoactive-Inotropic Score		17.0 ± 5.6	18.2 ± 5.7	.314
Number of children receiving postoperative peritoneal dialysis (n)		7 (21.9%)	8 (12.1%)	.338
HGB, g/L		145.5 ± 20.6	135.8 ± 26.6	.073
Albumin, g/L		37.8 ± 5.0	38.1 ± 5.2	.757
Prealbumin, mg/L		135.2 ± 29.5	131.0 ± 28.4	.549
RBP, g/L		18.9 ± 12.4	22.9 ± 7.0	.115

### 3.2 Comparison of the incidence of postoperative feeding intolerance in the 2 groups

The total incidence of feeding intolerance was 56.3% in 24-hour Group and 39.4% in 6-hour Group (*P* = .12). The occurrence of gastric retention and repeated trended to be higher in 24-hour Group as compared with 6-hour Group (*P* = .05 and .082, respectively). No serious feeding intolerance was found in either of the groups. The use of domperidone due to delayed gastric emptying after surgery was significantly reduced in 6-hour Group (*P* = .003) (Table 3).

**Table 3:** Comparison of feeding intolerance and use of prokinetic drug (domperidone) between the 2 groups.

Items	24-h group	6-h group	*P*
Number of patients with feeding intolerance (n)	18 (56.3%)	26 (39.4%)	.116
Number of patients with gastric retention (n)	9 (28.1%)	8 (12.1%)	.050
Number of patients with repeated vomiting (n)	9 (28.1%)	9 (13.7%)	.082
Number of patients with abdominal distension (n)	13 (40.6%)	17 (25.8%)	.134
Postoperative use of prokinetic drugs (n)	21 (65.6%)	22 (33.3%)	.003

### 3.3 Comparison of nutritional indicators at CICU discharge between the 2 groups

As compared to 24-hour Group, prealbumin and RBP were higher in 6-hour Group (*P* = .043 and .054). No significant difference was found in albumin levels (*P* = .33) (Table 4).

**Table 4:** Comparison of laboratory results when discharged from CICU between the 2 groups.

Items	24-h group	6-h group	*P*
Albumin, g/L	40.6 ± 4.7	41.6 ± 4.6	.326
Prealbumin (mg/L)	135.2 ± 28.9	160.7 ± 64.3	.043
RBP, g/L	23.0 ± 14.1	30.7 ± 17.7	.054

### 3.4 Comparison of outcome indicators between the 2 groups

The time from the initiation of EN to the termination of mechanical evacuation of the ventilator was set as the EN observation window time, and no significant difference was found between the 2 groups (*P* = .52). However, as compared to 24-hour Group, a significantly greater number of children reached the target calorie of EN at the window time (*P* = .04) in 6-hour Group. The durations of postoperative stay in CICU, total stay in CICU, postoperative hospital stay, and total hospital stay were significantly shorter in 6-hour Group (*P* < .05 for all). No difference was found in the postoperative ventilator-assisted ventilation time and the occurrence of ventilated-associated pneumonia between the 2 groups (Table 5).

**Table 5:** Comparison of various outcome indicators between the 2 groups.

Items	24-h group	6-h group	*P*
Incidence of VAP (*n*)	7 (21.9%)	10 (15.2%)	.410
EN observation window time (h)	77.2 ± 155.6	58.9 ± 37.4	.516
Number of patients reaching target calorie at the window time (*n*)	4 (12.5%)	21 (31.8%)	.040
Preoperative ventilator-assisted ventilation time (h)	18.0 ± 17.7	34.1 ± 23.4	.090
Postoperative ventilator-assisted ventilation time (h)	103.1 ± 162.6	65.7 ± 37.1	.208
Total ventilator-assisted ventilation time (h)	107.6 ± 163.5	76.1 ± 41.9	.290
Preoperative staying time in CICU (d)	4.1 ± 3.0	4.5 ± 4.3	.673
Postoperative staying time in CICU (d)	9.5 ± 10.1	5.6 ± 2.1	.040
Total staying time in CICU (d)	13.3 ± 10.4	9.4 ± 4.5	.049
Postoperative length of stay (d)	15.8 ± 10.3	11.6 ± 3.0	.028
Total length of stay (d)	19.7 ± 10.4	15.7 ± 4.4	.043
Hospitalization expense (d)	93565.5 ± 39958.3	83765.9 ± 15267.3	.189

## 4 Discussion

This study retrospectively analyzed data from 98 pediatric patients with complex CHD who met the inclusion and exclusion criteria within 3 years. Of these patients, 32 were in 24-hour group (initiation of EN 24 hours after surgery) and 66 in the 6-hour group (initiation of EN 6 hours after surgery). The comparison results between the 2 groups showed that the initiation of EN 6 hours after surgery was safe and feasible in children with complex CHD, which did not increase the incidence of feeding intolerance, but showed the duration of CICU and hospital stay and improving their systemic nutritional status, thus promoting their recovery.

The results of the present study suggest that the incidence of feeding intolerance was 56.3% in the 24-hour group and 39.4% in the 6-hour group, indicating that the initiation of EN 6 hours after surgery did not increase the incidence of feeding intolerance. This also effectively proves that early postoperative EN can shorten the time of postoperative intestinal paralysis, improve the microcirculation of liver and intestinal mucosa, and prevent the imbalance of intestinal flora, so it may better regulate gastrointestinal function, promote the recovery of gastrointestinal function, and reduce the incidence of feeding intolerance. Studies have shown the protective effects of small amounts of feeding on the gastrointestinal mucosa. The minimal enteral feeding should be maintained at least for neonates, rather than complete discontinuation of enteral feeding.^[24]^ The concept of ERAS has advocated very early initiation of EN after surgery, and related reports are available regarding its feasibility in different diseases in pediatric patients.^[16,17]^ The concept also states that patients undergoing abdominal surgery should be given a fluid diet and EN 6 hours after surgery, and then gradually transitioned to normal feeding. Open heart surgery does not belong to gastrointestinal surgery and there is no gastrointestinal anastomosis, so it is feasible to implement early postoperative EN theoretically. At present, no consensus exists for the specific time of initiation of EN, and more clinical studies are needed to provide evidence. The initiation of EN 6 hours after surgery did not increase the incidence of feeding intolerance, which was safe to a certain extent.

In children with gastric retention and lack of gastrointestinal motility, prokinetic drugs, such as domperidone, can be used. A study showed that the proportion of children receiving the administration of prokinetic drugs after cardiac surgery was 85%.^[25]^ The results of this study showed that the use of prokinetic drugs after surgery in the 2 groups of children, which was 65.6% in the 24-hour group and 33.3% in the 6-hour group, indicating the decrease in the use of prokinetic drugs for children in the 6-hour group. Early postoperative EN support therapy can shorten the time of postoperative intestinal paralysis, maintain the function of gastrointestinal mucosal barrier, facilitate the secretion of gastrointestinal hormones, promote the recovery of gastrointestinal function, and thus reduce the use of prokinetic drugs. The initiation of EN 6 hours after surgery, to a certain extent, could protect the gastrointestinal mucosa and promote the recovery of gastrointestinal function, which was beneficial for postoperative recovery in children.

Clinically, no gold standard exists for nutritional assessment,^[22]^ and the commonly used indicators include albumin, prealbumin, and RBP. Related studies suggested that the half-life of albumin was 14 to 20 days, which does not indicate immediate nutritional status, while the infusion of albumin, dehydration, sepsis, and other conditions affect albumin concentration. Prealbumin and RBP have short half-lives of 48 and 12 hours, respectively, and are good markers of visceral protein pools, but they cannot accurately reflect nutritional status under inflammatory and stress conditions.^[26]^ Only under stable clinical conditions, a meaningful nutritional assessment can be performed for the patients.^[27,28]^ In this study, albumin, prealbumin, and RBP were selected as nutritional indicators. The results of the present study showed that the prealbumin index was higher when the children were discharged from hospital, in the 6-hour group. Early EN is not only to provide nutrition, but more importantly to maintain the normal physiological functions of cells, organs and tissues, accelerate the recovery of tissues and organs, and thus improve the overall nutritional status of children and promote their recovery. When the condition of the children was stable, the 6-hour group had a higher prealbumin index, indicating that the nutritional status of the children had been improved to a certain extent.

The results of this study showed that the initiation of EN 6 hours after surgery could shorten their staying time in CICU and hospital. The main reason was that EN started in the early postoperative period, which resulted in faster recovery of gastrointestinal function and improved overall nutritional status of the children, so there were fewer complications and the recovery speed of the children was faster. Malnutrition may lead to some complications and increase ICU and hospital stay, hospitalization expenses, and mortality.^[29]^ Studies have shown that administration of less than 68 kcal/(kg.d) of energy for infants after cardiac surgery is associated with postoperative adverse events.^[30]^ The guidelines recommend that EN should be preferred if the child is tolerant, and energy should be supplemented while increasing the supply of protein and other nutrients.^[10]^ During the transition from fasting to total EN after cardiac surgery, 5% glucose solution can only be used to supplement energy. Although sufficient energy is provided, the supply of protein and other nutrients lacks to a certain extent, and EN can make up for this shortcoming. Children who receive more EN during the transition period obtain more comprehensive nutrients. In the present study, at the same observation window time, more children in the 6-hour group achieved the target calorie through TEN, obtaining a better nutritional supply.

In recent years, the initiation of EN as early as possible has been widely recognized. Comprehensive assessments are performed according to the age and gastrointestinal functions, and the energy, protein, vitamins, and minerals daily needed by children of all ages are supplied to improve their nutritional status, which plays an important role in the postoperative quick recovery of body function. The initiation of EN 6 hours after surgery was safe and feasible in children with complex CHD, which did not increase the incidence of feeding intolerance, but showed the duration of CICU and hospital stay and improving their systemic nutritional status, thus promoting their recovery.

## 5 Limitations

At present, little evidence exists to guide the initiation of EN in neonates with CHD.^[31]^ However, nutritional support for improved clinical outcomes is essential.^[4]^ This study was a historical control study with some limitations. Moreover, many clinical factors may interfere the results of the present study. It is hoped that randomized controlled trials will be conducted, and more rigorous processes will be designed to further validate the results of this study. Finally, the study compared the nutritional practice between 2 different periods, that is, 2015 versus 2016 to 2017. Medical cost increases gradually over the years. Although no significant difference in hospitalization expense was found in the present study, it is reasonable to estimate that the cost-and-benefit would be greater in the very early EN group given the shorter stay in the CICU and hospital.

## 6 Conclusions

The results of this study indicated that the initiation of EN 6 hours after surgery did not increase the incidence of feeding intolerance, but may promote the recovery of gastrointestinal function, reduce the use of prokinetic drugs, and improve the nutritional status and clinical outcomes.

## Acknowledgment

The authors thank the medical staff in the CICU of Guangzhou Women and Children's Medical Center for their assistance in collecting data.

## Author contributions

**Data curation:** Caixin Yin, Chen Gong, Xiuchun Chen.

**Investigation:** Na Du.

**Methodology:** Yanqin Cui, Wanhua Xie.

**Writing – original draft:** Na Du, Wanhua Xie.

**Writing – review & editing:** Yanqin Cui.
